# 

**Published:** 1869-04

**Authors:** 


### INDEX.

### Volume V to Volume VIII.

PAGE.

**COMMUNICATIONS.**

**A** Drop of Water, Prof J D Steele, - - 12
An Essay on Boils, A Job - - - - 16
A Law of Cure, P H Hayes, M D - - - 31
A Birth and a Death, Dr Ned, - - - - 85
A Word concerning Doctors, B H Pratt, A M - 211
**B**ones, Ishmæl, - - - - - - 107
**C**hildren's Food, Uncle Bob, - - - 294
Child Nature, P H Hayes, M D - - 266
Cutting the Nerves, P H Hayes, M D - - 88
**D**ress, from the Health Point, B H Pratt, A M 55
Drafts, B H Pratt, A M - - - - - 264
**E**mployment for Children, Uncle Bob, - 240
**F**amily Covernment, " " - - 113
**H**ousekeeper's Department, Housekeeper, 21--59
House Building, Ben H Pratt, A M - - 133
How to Choose a Doctor, Thos K Beecher, - 163
**L**iver, Dr Ned, - - - - - - 111
Light, P H Hayes, M D - - - - - 238
Life Insurance, Thos K Beecher, - - - 263
Little Helpers, Uncle Bob, - - - 267
**M**edical Reform, B H Pratt, A M - - 237
**O**f Springs, Thos K Beecher, - - 17
On Boys, Uncle Bob, - - - - - 87
**P**rodigy Making, B H Pratt, A M - - 13
Pen Wipings, Robt A Hall, - - - - 18
Profit and Pleasure. Thos K Beecher, - 185
Phrenologic, B H Pratt, A M - - - 186
**Q**uack Doctors, W C Coleman, M D - - 295
**S**leeplessness, B H Pratt, A M - - - 159
Stomach Ache and Consumption, Thos K Beecher, 213
**T**rading and Shopping, Robt A Hall, - 32
The Stomach, Asthetically considered, Ishmæl, 57
The Help Question, B H Pratt, A M - 81--108
The Stomach, Familiarly Treated, Ishmæl, - 84
The Skin, Ishmæl, - - - - - 136
Train up a Child, B H Pratt, A M - - 291
Teasing Children, Uncle Bob, - - - 188
Tea Tasting, P H Hayes, M D - - - 293
**U**terine Displacements, L A Babcock, M D, L L D 114
**W**hat to Eat, B H Pratt, A M - - - - 29
What are Children Good For, Uncle Bob, - 137
Why, Uncle Bob, - - - - - - 162

**EDITORIAL,**

**A** Home Conversation. - - - 6
About Doctors, - - - - - 44
Appetite, - - - - - - 45
American Quackery, - - - - 71
A Precious Swindler, - - - - - 126
Abuse of the Eyes, - - - - 173
Artificial Pupil, - - - - - 229
An Aerial Voyage, - - - - 280
A New Swindle, - - - - - 175
Apology, - - - - - - 306
**B**e Careful, - - - - - - 16
Business Notes, - - 23-51-78-103-130-210
Babies, - - - - - - - 42
Books, Magazines and Periodicals, 20-48-53-70-102-132-148 181-234-259-287-209-311.
**C**ancer Quacks, - - - - - 9
Care of Babies, - - - - - 73
Consumptive Children, - - - - 124
Cancer Doctors, - - - - - - 152
Carpets vs Health, - - - - - 174
Cataract, - - - - - - - 199
Croup, - - - - - - - 202

PAGE.

Color Blindness, - - - - - 204
Children's Winter Dress, - - - - 204
Close of Vol VII - - - - - - 306
**D**omestic Medicine, 9-49-67-99-146-171-194-222-249-277-303
"Doctor," - - - - - 204
Diagnosis, - - - - - - - 251
Diseases of House Painters, - - - 73
Dyspepsia, - - - - - - - 252
**E**ditorial Chit Chat, 4-45-76-96-128-154-178-206-231-255 285-308.
Education of Children, - - - - 228
Eyelashes, - - - - - - - 253
**F**idgets, - - - - - - - 198
Fire, - - - - - - - - 251
**G**ranular Lids, - - - - - 149
**H**ints to Mothers and Nurses, - - - 25
Hair Restorers. - - - - - 43
Have a Cigar, sir? - - - - 101
Hay Fever. - - - - - - 150
**I**ngrowing Eyelashes, - - - - 72
Injuries to the Eye, - - - - - 125
Intermarriages, - - - - - 182
**M**edical Items and News, 7-33-61-89-117-138-164-189-215-241-269-
Medical Staff Rank, - - - - 122
Meddling with the Ears, - - - - 175
Milk and Lead Poisoning, - - - - 182
Medicine vs Advice, - - - - - 202
Medical Journals and Patent Medicines, - - 203
Meat, - - - - - - - 253
**N**ewspaper Receipts, - - - - 127
New York Swindlers, - - - - - 230
**O**varian Tumors, - - - - 226
**P**robing and Syringing the Ear, - - 19
Position in Chloroform, - - - - 20
Poisonous Hair Dyes, - - - - 176
Premiums, - - - - - 183-263
**Q**uack Medicines, . - - - - 33
**R**est, - - - - - - - 41
Rheumatism, - - - - - 126
Revaccination, - - - - - - 255
Reading in the Cars, - - - - 307
**S**windlers, - - - - - - 24
Scarlet Hose, - - - - - - 105
Sixth Volume, - - - - - - 122
Self Styled Doctors, - - - - - 123
Sun Baths, - - - - - - 151
Seventh Year, - - - - - - 226
Sympathetic Affections of the Eye, - - 283
Strychnia in Beer, - - - - - - 11
Summer Eating, - - - - - - 43
**T**he Eye, - - - - - - - 3
To Correspondents, - - - - 19
The Bistoury for 1870, - - - 96
The Bistoury Given Away, - - - 184
Time's Up, - - - - - - - 199
The Envelope, - - - - - - 199
Trusses, - - - - - - - 205
The Blind Spot, - - - - - - 205
Transplanting the Skin, - - - - 235
To Physicians, - - - - - - 279
Traveling Impostors, - - - - - 284
**W**e make a Bow, - - - - - 3
Woman's Rights, - - - - - 74
We Grow, - - - - - - - 41
What to Eat, - - - - - - - 153

PAGE.

**CORRESPONDENCE.**

Six Letters with Answers and Advice, - 26
Answers to Correspondents, 28-54-80-106-132-158-182-211-235-301
Letters Exposing Humbugs, - - - 39
Letters on Swindlers, - - - - - 66
Letter in Answer to Ishmæl, - - 93
Letter from a Wife and Mother, and other Letters, 93
Letter Exposing Sayer & Co. On Tape Worm, From "A Wife." From U S Counsel at Brunswick, Germany, etc, - - - - 120
Complimentary Letters and those that are not, Letter from Germany and Scotland, together with others of interest, - - - 142
Letter Exposing Rev (?) Chas E King, Letter on Tape Worm, etc, - - - 168
Letters Exposing Jos T Inman and other Humbugs, 192
Letter Exposing M Wagner & Co, and their "Sunlight Oil," Laying-on-of-hands Chap and other Swindlers, - - - 219
Letter Exposing S R Shaw, with other Swindling Concerns, - - - - - 247
Letters and Answers to Correspondents, - 274
Letter from Phila. Quack College, with other letters, 302

**CLIPPINGS.**

A Clerical Surgeon, - - - 16
A Case of Petrefaction, - - - 37
A Precocious Child, - - - 60
A Stick of Candy, - - - - - 103
Ague, to Avoid - - - - - 279
Bad Wine and Whisky, - - - 37
Bleaching Sponges, - - - 60
Benefits of Laughter, - - - - 308
Copious Prespiration, - - - - 22
Count Moultke - - - - - 222
Chilblains and Chapped Hands, - - 224
Death and the Devil, - - - 154
Dentists, - - - - 198
Effects of Tight Lacing, - - - - 11
Fate of Io, - - - - 22
Fools vs Imps, - - - - - 241
Formulæ for Druggists, - - - 245
Fun and Farming, - - - - - 268
Giants, - - - - - - 154
Genuine Port Wine, - - - - 174
Good News if True, - - - - 177
How to Cure a Cold, - - - - - 177
How to make Farm Life Attractive, - 219
Hœmorrhoids, - - - - - 235
How much to Eat, - - - - 260
Inferiority of our Coffee, - - 104
Insects as Food, - - - - - 197
I Say, Captain, - - - - 188
Infant Feeding, - - - - 262
Infallible Remedies, - - - 279
Infant Monstrosity, - - - 288
Japanese Domestic Life, - - 172
Jewish Physicians in Rome, - - - 241
Longevity in Mexico, - - - 16
Lomi-Lomi, - - - - - - 196
Natural Steam Baths, - - - - 105
Old Folks, - - - - - - 152
Often Done—Poetry, - - - - 288
Poisonous Socks, - - - - - 43
Peas and Beans, - - - - 93
Poisonous Effects of Orange Peel, - - 198
Pompaiian Statutes, - - - 261
Painting Cemented Walls, - - - 262
Protection for Wet Weather, - - 276
Quarterly Report, - - - 53-80-106-289
Quack Chemists, - - - 225
Sweedish Railways, - - - - - 37
Strange Freak of Nature, - - 58

PLGE.

Sewerage Manuring, - - - - 174
Sleeplessness in Infants, - - - 176
Suicide, - - - - - - 197
Suez Canal, - - - - - 279
Tributes to Vanity, - - - 37-65
The Vehicle, - - - - - 40
The Doctor, - - - - - - 260
Tattooed, Interesting Case, - - 305
Vine Culture in France, - - - 182

**DOMESTIC MEDICINE.**

Containing valuable hints for treatment of ordinary ailments, together with Receipes of use in every Household.

Abortion, - - - - - 68
Artificial Respiration, - - - - 68
Anointing in Disease, - - - - 171
Black Eye, - - - - - - 9
Broken Bones, - - - - - 9
Burns and Scalds, - - - - 49-171
Bathing, - - - - - 50
Bowel Complaints, - - - - 69-147
Bleeding, to Arrest - - - 146
Blisters, Treatment of - - - 171
Beef Tea, - - - - - 194
Bay Rum, How to make, - - - 249
Bad Breath, - - - - - 250
Beets, How to cook - - - - - 277
Birds, " " - - - - - 277
Broil without Burning, - - - - 277
Bruises in Furniture, - - - - 278
Beds, to Air - - - - - 278
Baldness, Remedy for - - - 303
Croup, - - - - - - 10-171
Cough Mixture, - - - - 11-194
Constipation, - - - - 49-249
Cholic in infants, - - - - 49
Cholera Mixture, - - - - - 51
Cough, to Allay - - - 67-224-277-
Corns, - - - - - 67-222
Complexion, to Beautify - - 67
Catarrh, - - - - - 67-100
Chapped Hands and Lips, - - 99-303
Carbuncle, - - - - - - 99
Cement for Preserve Jars, - - 172
Cleaning Silver Ware, - - - 195
Consumption, - - - - - 249
Cologne, To make - - - - - 303
Diarrhœa in Children, - - 50-249-277
Dyspepsia, - - - - - - 147
Diabetes, - - - - - - 194
Deadly Cheese, - - - - - 223
Dandruff, - - - - - 249
Ears, Care of - - - - - 69-100
Eyes, Infants, - - - - - 100
Erysipelas, - - - - - - 223
Eggs, To Preserve - - - - 303
Felons, - - - - - - 10
Fever and Ague, - - - - - 171
Flies, to Destroy - - - - - 172
Florida Water, - - - - - 249
Freckles and Tan, - - - - 249
Feeding Bottles, - - - - - 277
Gums, Disease of - - - - - 49
Gums, Lancing - - - - - 69
Glanders, - - - - - - 195
Gloves, To Clean - - - - - 196
Green Veils, - - - - - - 223
Grease, To Remove - - - - 303
Hair, Falling off - - - - - 50
Hernea, - - - - - 146
Hair Dyes, - - - - - - 223

| | PAGE. |
|---|---|
| Itch, | 171 |
| Indelible Ink, | 195 |
| Jam Preserving, | 304 |
| Measles, | 10 |
| Mumps, | 222 |
| Mustard Poultices, | 67 |
| Marry, When to | 68 |
| Marble, to Polish | 278 |
| Mucilage, | 303 |
| Nail, Ingrowing of | 99-171 |
| Nose Bleeding, | 222 |
| Neuralgia in Face, | 223 |
| Piles, | 59-304 |
| Pin Worms, | 68-304 |
| Pessary, | 100 |
| Paint, To Clean, | 172 |
| Poison by Ivy, | 223 |
| Paste for Tin, | 277 |
| Polish for Linen, | 278 |
| Quinine, How to take it, | 146 |
| Quinsy, | 146 |
| Ringworm, | 10-172 |
| Rat Bites, | 99 |
| Rusty Nails, Injury from | 99 |
| Rupture, | 194 |
| Roaches, To get rid of | 195 |
| Rheumatism, | 222 |
| Scrofulus Swellings, | 10 |
| Sore Throat, | 10 |
| Sore Eyes of Infants, | 11 |
| Soap, To make | 303 |
| Sun Stroke, | 49-147 |
| Salts, How to take | 67 |
| Salt-Rheum | 171 |
| Spasms, | 194 |
| Shoulder Braces, | 195 |
| Skin, Inflammation of | 222 |
| Sea Sickness, | 250 |
| Spots on Mahogany, | 277 |
| Small Pox, | 277 |
| " " To prevent Pitting from | 303 |
| Sweet Pickles, To make | 278 |
| Sunshine in Sickness, | 278 |
| Sunflower Seeds, | 304 |
| Typhoid Fever, | 69 |
| Tape Worm, | 99-194 |
| Tomato Vinegar, | 172 |
| Teeth, Decayed | 195 |
| Toothache, | 222 |
| Whooping Cough, | 11-99 |
| Worm Tea, | 49 |
| Winter Sports, | 99 |
| Winter Clothing, | 100 |
| Worms in Children, | 147 |
| Waterproof Paper, | 172 |
| Washing Fluid, | 196 |
| Weather Glass, | 250 |
| Warts, | 277 |
| Wall Paper, Sickness from | 278 |

**MEDICAL ITEMS AND NEWS,**

Containing the Latest and most Reliable Formulæ for Treating all manner of Disease Culled from the Standard Medical Journals of the World; carefully arranged by the Editor.

| | PAGE. |
|---|---|
| Amaurosis from Crowding Teeth, | 7 |
| Abortion, | 33-89 |
| Anurism, Poplitial, | 36 |
| " Carotid | 62 |
| Anilin, Sulphate of | 118 |
| Anointing in Disease, | 138 |
| Ague, Carbolic Acid in | 139 |
| Anæsthesia, | 140-216 |
| Anæsthesia, Local | 300-301 |
| Anatomical Specimens, To Preserve | 189 |
| After Pains, | 190 |
| Asthma, | 190-218 |
| Anæmia, | 269 |
| " Idiopathic | 242 |
| American Medical Association, | 269 |
| Aural Catarrh, | 243 |
| Acne, | 244 |
| Abcess, Mammary | 244 |
| Albuminuria, | 244 |
| Alcohol, To Test, | 297 |
| Anus, Fissure of | 298 |
| Boils, Whitloes and Abscesses, | 7 |
| Blennorrhœa and Lucorrhœa | 34 |
| Bronchitis and Phthisis, | 35 |
| " in Children, | 63 |
| " Infantile, | 216 |
| Burns, | 117-139-271 |
| Bath, Turkish, | 117 |
| Belladonna Plaster, | 119 |
| Blindness from Intermittent Fever, | 191 |
| Biliary Calculi, | 216 |
| Bromide of Potassium for Children, | 217 |
| Brown-Sequard, Dr. | 241 |
| Bullet in Brain 19 years, | 272 |
| Bronchocele, | 298 |
| Chloroform, to prevent death from | 8-119-140-164 |
| Catarrh, | 34 |
| " of Bladder, | 34 |
| Cataract, treatment for | 34 |
| " fee for | 62 |
| Cod Liver Oil, | 61 |
| Chlorosis, | 61 |
| Cadavar, To preserve | 61 |
| Constipation in Old Age, | 62 |
| Chancres, | 63-138 |
| Caries, Carbolic Acid in | 90 |
| Cervex Uteri, How to Cauterize | 90 |
| Chorea, | 90-117-215-218-272-297 |
| Croup, | 120-272-273 |
| Chloral Hydrate, | 141-191-215 |
| " " Amputation in | 138 |
| " " and Cod Liver Oil, | 269 |
| Cancer, | 139-164 |
| Constipation, Habitual | 165-192-217-298 |
| Correction in formula, | 166 |
| Cod Liver Oil, Digestion of | 190 |
| " " " and Iodide of Iron, | 299 |
| Colorless Tincture Iodine, | 190 |
| Coughs, | 191 |
| Cholera Infantum, | 191-269-300 |
| " Morbus, | 216 |
| Cancrum Oris, | 217 |
| Cancer of Liver, | 217 |
| Cerebral Disease, | 242 |
| Condurango, | 269 |
| Convulsions, Puerperal | 269 |
| Cathartic Pill, | 271 |
| Carbolic Cerate, | 271 |
| Calculus Nephritis, | 300 |
| Carbolic Acid, | 301 |
| Diarrhœa, | 35-63-117 |
| " in Infants, | 34 |
| " Wet Sheets in | 299 |
| Dressing for Wounds, | 34 |
| Diabetes, | 35-89-119-165 |
| Delirium Tremens, | 62-120-167-215 |
| Duodenitis, Chronic | 62 |
| Dysmenorrhœa, | 63-141 |
| Dover's Powder, Substitute for | 63 |

| | PAGE. |
|---|---|
| Dysentery, | 64-189 215-269 274-297 |
| Diptheria, | 139-241-273-299 |
| Dyspepsia and Oxaluria, | 164 |
| Depilatory, | 189 |
| Double Headed Child, | 215 |
| Dilution of Lymph, | 241 |
| Dahlberg's Tincture, | 297 |
| Epilepsy, | 63-89-117-141-164-245-273 |
| " from carious teeth, | 7 |
| " of the Retina, | 191 |
| " Infantile, | 242 |
| Ergot, as a hæmostatic, | 164 |
| Epithelial Cancer, | 164-269 |
| Enlarged Tonsils, | 165-242-298 |
| Elixir of Chloroform, | 166 |
| " Calasaya and Iron, | 245 |
| Erysipelas, | 189-297 |
| Eczema, | 215 |
| Fever, Malarious | 34 |
| " Intermittent, | 61-189-270 |
| " Puerperal, | 91-215 |
| " Typhoid, | 139-243-270-300 |
| " Rheumatic, | 166 |
| " Typho-cerebral, | 166 |
| " Scarlet | 64-191-215 |
| " " Gargle for | 192 |
| " Yellow, | 297 |
| Fistula in Ano, | 244 |
| Frozen Beef Essence. | 299 |
| Glycerine, Preparations of | 7 |
| Gonorrhœa, | 8-63-189 270-272 |
| Glandular Tumors, | 35-190 |
| Gleet, | 35 |
| Goitre, | 63 |
| Glycerine of Tannin, | 117 |
| Gout, | 140 |
| Gastric Douche | 242 |
| Gangrene, | 298 |
| " Senile | 271 |
| Hydrocephalus, | 61 |
| Hæmorrhoids, | 90-119-217-298 |
| Hemicrania, | 117 |
| Hysterical Aphonia, | 118 |
| Hypodermic Doses of Medicine, | 167 |
| Hysteria, | 189 |
| Hiccough, | 215 269 |
| Headache, Nervous | 216 |
| " Hysterical | 216 |
| " Turpentine in | 270 |
| Hæmoptysis, | 270 |
| Hæmorrhage and Ipecac, | 298 |
| Indurated Hæmorrhoids, | 33 |
| India Rubber Sponge, | 33 |
| Indigestion, | 90-189-217 |
| Iron Stoves, | 117 |
| Indolent Condylomata, | 118 |
| " Ulcers, | 216 |
| Iritis, Syphilitic, | 139 |
| Iodoform, | 189 |
| " Ointment, | 271 |
| Insomnia, | 216 |
| Intestines, Obstruction of | 218 |
| Incontinence of Urine, | 92-243-269 |
| Lung, Amputation of | 61 |
| Lupuline, | 119 |
| Leucorrhœa, | 215 |
| Malignant Pustule, | 9-119-190 |
| Milk Diet, | 119 |
| Menorrhagia, | 140 |
| Mineral Waters, | 247 |
| Measles, | 273 |
| Medical Director, | 297 |
| Nervous Headache, | 8-34-89-216 |
| New Anæsthetic, | 34 |
| Neuralgia, | 63-140-164-192-297 |
| " Facial, | 90-119 |
| " Ovarian, | 139-190 |
| Nævus, | 141-190 |
| Nausea from Opium, | 241 |
| Nephrotomy, | 271 |
| Opium and Stramonium, | 8 |
| Ovariotomy at 12 Years, | 61 |
| Ophthalmia, Strumous. | 91 |
| " Purulent, | 35 |
| Opium Eaters, | 117 |
| Ozæna, | 117-215 |
| Oxygen, Inhalation of | 118 |
| Os Uteri, Ulceration of | 141 |
| Oxaluria, | 191 |
| Odontalgia, | 215 |
| Ozone in Phthisis Pulmonalis, | 243 |
| Oakum, | 269 |
| Otorrhœa, | 270 |
| Pertusses, | 7-34-118-140-244-273 |

| | PAGE. |
|---|---|
| Polypus, Nasal | 8 |
| Poisoning by Mushrooms, | 8-33 |
| " " Phosphorus, | 7 |
| " " Lead, | 33 |
| " " Morphia, | 89 |
| " " Opium, | 117 |
| " " Ergot, | 7 |
| Poison Oak, | 244 |
| Puerpura, | 8 |
| Prurigo, | 34-164 |
| Pruritus Vulvæ | 34-140 |
| Purulent Ophthalmia, | 35 |
| Prolific Child Bearer, | 61 |
| Painless Surgery, | 118 |
| Phthisis, | 119 |
| " Pulmonalis, | 243-299 |
| Psoriasis, | 120 |
| Pneumonia | 168-217 |
| Phthisical Sweating, | 189 |
| Peurperal Convulsions, | 189 |
| Pleurisy, | 218 |
| Pay of Army Surgeons, | 242 |
| Peritonitis, | 300 |
| Quaint Prescription, | 62 |
| Quinine, Sweet | 118 |
| " How to Give | 118 |
| " Hypodermically | 164 |
| " To Disguise | 215 |
| Rheumatism, | 36-62-64-92-241-271 |
| Sponge Tent, | 7 |
| Syphilis, | 35-61 |
| " Tertiary, | 62-89 |
| " Infantile, | 90 |
| " Subcutaneous Medication of | 118 |
| " Headache in | 164 |
| " and Scrofula, | 218 |
| " Dermato, | 297 |
| Sciatica, | 35 |
| Sunstroke, | 36 |
| Sutures, | 61 |
| Spina Bifida, | 64 |
| Scarlet Fever, | 64 |
| Scrofula, | 89 |
| Spinal Irritation, | 90 |
| Suppositories, | 90-243 |
| Soup, Liebig's | 139 |
| Sweating Feet, | 139 |
| Singular Diet, | 139 |
| Sleeplessness. | 139 |
| Sæsarian Section, | 140-242 |
| Suppuration, | 165 |
| Stomach, How to empty | 165 |
| " Disease of | 192 |
| " Irritability of | 216 |
| Styptic Paper, | 166 |
| Softening of the Brain, | 190 |
| Sea Sickness, | 218 |
| Smallpox, | 242-297-298-299 |
| " Pitting in | 269 |
| Syrups for Soda Fountains, | 245 |
| Secondary Lymph, | 270 |
| Summer Complaint, | 272 |
| Scabies, | 297 |
| Santonin, To dissolve | 300 |
| Tobacco, Influence of | 8 |
| " Denicotinized | 91 |
| Trismus Neonatorum, | 8-117 |
| Tubercle, Prevention of | 9 |
| " Arrest of | 119 |
| Typhoid Fever, | 33 |
| Tape Worm, | 62-89 |
| Tetanus, | 117 |
| Tooth Ache, | 120 |
| Transfusion of Blood, | 140-166 |
| Tar Water, | 165 |
| To Detect Lung Tissue, | 189 |
| Tenesmus, | 243 |
| Tympanic Distention, | 298 |
| Ulcers, Eczema, etc. | 35 |
| Uterus, Exterpation of | 118 |
| Uterine, Hemorrhage, | 190 |
| Venerial Ulcers, | 33 |
| Varicocele, | 91 |
| Vaginismus, | 118 |
| Vomiting in Phthisis, | 165 |
| " Pregnancy, | 190 |
| Velpau's Mixture, | 242 |
| Varicose Ulcer of Leg, | 269 |
| Whooping Cough. | 34-118.140-244-273-297-298 |
| Warts, Syphilitic. | 89 |
| Wounds, Irrigation of | 91 |
| Wine of Beef and Iron, | 299 |
| Young Mother. | 8 |
| Yellow Fever, | 297 |

### To Correspondents.

We invite our subscribers to write to us for advice relative to their ailments, or any other subject of interest to them, and we will answer them if we can, or find some person who is "better posted" than we are to do it for us.

If you intend your letters for publication, write upon but one side of the paper, and make them as short as possible.

If you require an answer "by return mail," don't forget to enclose a postage stamp to pay postage, otherwise you will be required to wait until the next issue of the BISTOURY for your answer.

### Answers to Correspondents.

Under this head we will answer all communications addressed to us of interest to the general reader, and will reply to such persons who forget to inclose us a postage stamp.

We will cheerfully give our advice in this department, to all who seek it, and solicit correspondence upon all subjects of interest to the people generally.

J. T., *Canton. Pa.*—For treatment of a "felon," see Domestic Medicine Department.

Mrs. C., *Port Jervis, N. Y.*—Wants to know how she can tell "whether she has an ovarian tumor or not."

Employ some skillful surgeon to ascertain.

SAMUEL WRIGHT, *Sigourney, Iowa.*—You must be more explicit in describing your symptoms. We do not understand you.

Dr. S., *Phillipsville, Ala.*—A sphygmograph may be had of Geo. Tiemann & Co., 67 Chatham street, N. Y., at a cost of about $40.

Dr. ——, *Randolph, Ga.*—You did not cut the muscle sufficiently. Try again—the eye must become straight, if you do your work properly.

Dr. J., *Anderson, Tenn.*—A first class artificial leg can be had in N. Y. for $100. Wm, H. Gregg & Co., Wholesale Druggists, of this City, can furnish you medicines as cheap as you can procure them in New York.

R. S. *Perue, Ind.*—We have never examined Rev. Milburn's eyes; cannot say what the trouble is, or whether he is curable or not. He is at present under the care of VON GRAFE, at Berlin; but has not yet received any benefit.

Mrs. A. L., *Binghamton, N. Y.*—Use a weak solution of *Carbolic Acid*—about ten grains in a pint of water. Apply with a syringe morning and evening. You will find it an excellent application for ulceration of the uterus, and the offensive discharge you complain of.

Dr. N., *Barnwell.* S. C.—The *Record* published by Wm. Wood & Co., N. Y., at $4 per year is an excellent journal. It is what you require.

Tiemann & Co. can furnish you with the instruments mentioned.

Mrs. R., *Troy, N. Y.*—"Fumigators," "Catarrh Snuffs," and all that sort of thing, are productive of great injury, while they can do no good whatever, in the treatment of catarrh.

The October number of the BISTOURY gives a plan of treatment for this difficulty.

F. L. R., *Middlesex, N. J.*—A hard tumor growing rapidly in the breast is a very dangerous thing. The breast should be removed immediately, before th glands in the axilla become involved. This is the only chance for cure. Avoid plasters and "cancer doctors," if you value your life.

S. R. M., *Cincinnati, Ohio.*—Prolapse of the rectum can be remedied by means of a surgical operation. A fold should be removed from either side of the prolapsed portion, by means of ligatures. This will lessen the calibre of the bowel and prevent its falling down. You would be required to remain here a month.

L., *Canandaigua, N. Y.*—It is a dangerous practice to syringe a child's ear. No good can result from such a procedure for ear-ache, while harm might be done the drum, if the water is thrown against it with any force. Procure equal parts of pure *glycerine* and *tincture of opium*, and drop a few drops in the ear, confining it with cotton. If this does not stop the ache, consult a surgeon, and learn what the difficulty is.

### Our Exchanges.

[*The following notices are not "puffs." We mention only such Journals as are really worthy of patronage.*]

HARPER—the Weekly, Magazine and Bazar, three of the best publications in America, have reached our table. We know of no way of expending ten dollars more usefully than to enclose that amount to the Harper Brothers, New York, and secure all three of these delightful periodicals.

PETER'S MUSICAL MONTHLY is a very handsome Magazine, containing new and beautiful songs and music, that are worth many times the subscription price—$3,00 per year. Send to J. L. Peters, 198 Broadway, for a copy.

PETERSON—is always welcome. It grows in favor monthly with the ladies, and gives, for two dollars a year, better fashion plates, designs and reading matter than other Journals charge from three to five dollars for. Chas. J. Peterson, 306 Chestnut street, Philadelphia, Pa , is the address.

No. 9, of "Zell's Popular Encyclopedia and Universal Dictionary," has reached us. The Editor is expending a vast amount of labor upon it, and we predict for it a popularity that will establish it a place in the library of every man in the country. It is the very cheapest mode of purchasing a reliable Universal Dictionary that we know of. It is issued in weekly numbers at ten cents per number. Enclose $1,00 to the publisher, and receive the first ten numbers, you will then be sure to become a subscriber to the end of the series.

T. Ellwood Zell, Nos. 17 and 19 South 6th street, Philadelphia.

READABLE PAPERS.—The *Corning Journal* and the *Williamsport Bulletin* come to us regularly, marked "BISTOURY." We return thanks therefor and desire to assure their Editors that they are two of the best weeklys that reach our sanctum.

OUR HOME JOURNALS.—The *Advertiser* has increased its circulation and its interest by establishing correspondents in all our neighboring towns. It has never been so readable as now—has the right sort of men to edit and manage it—is rapidly taking the place of the New York papers in this section of country, and has shown itself worthy of success by its enterprise.

The *Gazette* is as gay and chatty as ever—we could'nt get along without it. As a local paper it has no superior in this part of the country. CHARLEY knows how to make his locals spicy and interesting. Subscribe for his paper and see.

The *Saturday Evening Review*.—We received our first copy last Saturday night. Boy brought it to our door—we took it in—opened it—Whew! Gracious! what a splendid sheet, we exclaimed." "Perfectly splendid," says wife.—"O, is'nt it nice, can read it without my spectacles," says Grandma. "O, Pa! see the Bramas there!" exclaims our nine year old who had a glimpse of the Poultry Department, and even baby came toddling up and expressed his admiration for the new family paper, by clapping his little hands and crowing merrily. Soon the entire household was interested to "know all about it," so we read it through, aloud, for them, and then all voted that it should be a regular visitor at our house.

The typographical appearance of the *Review* far surpasses any thing of the kind ever brought to this city, while the editorial and selected matter is calculated to interest and instruct almost every member of your household.

WHEELER & WATTS, Editors and Proprietors. Terms $3,00 per year. Send for a specimen copy.

---

LOVE VS. BEEF.—A bachelor uncle, to whom his niece applied for good advice on the question of choosing between two suitors, one of whom was rich and the other poor—the latter, of course, being the most ardent as well as favored suitor—sententiously replied: "My dear, the question being stripped of all illusory elements, your choice simply lies between love and beef. Now love is an idea, and beef is a reality. Love you can get along without, but beef you must have. Therefore make sure of your beef."

---

EFFECTS OF TOBACCO.—The usage of smoking tobacco by children causes paleness, loss of flesh, palpitation of the heart, derangement of the digestion, and all the symptoms of impoverished blood. No treatment is effectual as long as the habit is continued. The intelligence is diminished, and a tendency to strong drink excited. These symptoms disappear where there is no organic lesion, with the discontinuance of the habit.

